# Successful Diagnosis and Treatment of Early Postpartum Choriocarcinoma

**DOI:** 10.1002/cnr2.70328

**Published:** 2025-08-28

**Authors:** Zeqing Du, Luyang Su, Sufen Zhao, Shizhao Wang

**Affiliations:** ^1^ Department of Obstetrics and Gynecology The Second Hospital of Hebei Medical University Shijiazhuang China; ^2^ Physical Examination Center Hebei General Hospital Shijiazhuang China; ^3^ Department of Anesthesiology The Second Hospital of Hebei Medical University Shijiazhuang China

**Keywords:** gestational trophoblastic tumor, persistent postpartum hemorrhage, postpartum placental choriocarcinoma, β‐hCG

## Abstract

**Background:**

Postpartum placental choriocarcinoma is a rare gestational trophoblastic tumor, with an incidence of approximately one in 50 000. Patients often present with persistent postpartum hemorrhage, which can lead to delayed diagnosis, hematogenous metastasis, and ultimately, a poor prognosis.

**Case:**

A 35‐year‐old woman was admitted to the Emergency Department 39 days after a cesarean section due to persistent heavy vaginal bleeding. Initial evaluation revealed a serum β‐human chorionic gonadotropin (β‐hCG) level of 7868 mIU/mL, transvaginal ultrasound identified a vascularized uterine mass with myometrial invasion, and MRI suggested residual tissue implantation. Following exclusion of retained products of conception, postpartum choriocarcinoma was suspected. The patient underwent five cycles of FAV chemotherapy (fluorouracil + actinomycin D + vincristine), resulting in undetectable β‐hCG levels and resolution of imaging findings after two cycles. Three additional consolidation cycles were administered, with complete remission confirmed at the final follow‐up.

**Conclusion:**

Pregnancy‐related causes must always be ruled out in women of childbearing age who present with irregular vaginal bleeding, even during the postpartum and lactation periods. In patients with postpartum bleeding, vigilance should be heightened to rule out the possibility of a pregnancy‐related Sertoli cell tumor.

## 1 Introduction

Postpartum choriocarcinoma, a highly aggressive gestational trophoblastic neoplasm (GTN), is an uncommon but serious complication of pregnancy, with an estimated incidence of approximately one in 170 000 births. This rare disease, characterized by abnormal trophoblast proliferation, poses a unique challenge to obstetricians and gynecologists worldwide [1]. Symptoms, such as irregular vaginal bleeding, may appear months after delivery [2]. Given its aggressiveness and potential for rapid metastasis, early diagnosis and prompt treatment are essential to ensuring a favorable prognosis for patients. Recent studies highlight persistent challenges in timely diagnosis, with misdiagnosis rates exceeding 90% in some cohorts, primarily due to nonspecific symptoms and delayed clinical suspicion [2, 3]. Notably, Soper [4] emphasized that β‐human chorionic gonadotropin (β‐hCG) monitoring remains the cornerstone of GTN management, even in the absence of histopathological confirmation. Emerging evidence further suggests that integrating advanced imaging (e.g., MRI with diffusion‐weighted sequences) may enhance early detection of myometrial invasion [5].

Several studies have highlighted the critical role of serum β‐hCG monitoring in the detection of postpartum choriocarcinoma [6, 7, 8]. As a tumor marker, β‐hCG levels tend to be persistently or inappropriately elevated after delivery in choriocarcinoma patients, serving as an early indicator of the disease [2]. Additionally, the combined use of multiple diagnostic methods, including pelvic ultrasound and radiological examinations, has been shown to improve diagnostic accuracy and facilitate early intervention [3, 9].

Despite advances in diagnostic techniques, postpartum choriocarcinoma remains a diagnostic challenge, often leading to delays in treatment. Misdiagnosis rates of up to 90% have been reported, largely due to the nonspecific and rare symptoms of the disease. This underscores the need for heightened vigilance among healthcare providers, particularly when managing postpartum patients presenting with irregular vaginal bleeding. This study aims to contribute to the existing body of knowledge by reporting a successful case of early diagnosis of placental choriocarcinoma following a cesarean section. By detailing the clinical course, diagnostic approach, and treatment outcome of this patient, we emphasize the importance of early intervention in improving the patient's prognosis. Timely initiation of multi‐agent chemotherapy resulted in rapid normalization of serum β‐hCG levels and resolution of imaging lesions. Despite advances in treatment, postpartum choriocarcinoma remains underdiagnosed due to its nonspecific presentation, with misdiagnosis rates exceeding 90% in some cohorts. This highlights the critical need for integrating β‐hCG monitoring and interdisciplinary collaboration in postpartum care, particularly in cases of unexplained hemorrhage.

This study is unique in its focus on a rare and often under‐recognized complication of pregnancy. By presenting successful treatment outcomes, we aim to raise healthcare professionals' awareness of the importance of early diagnosis and prompt treatment of postpartum choriocarcinoma. Furthermore, our case report contributes to the limited literature on this topic, providing valuable insights into diagnostic strategies and treatment approaches.

## 2 Clinical History

A 30‐year‐old woman presented to the Emergency Department on March 30, 2024, with sudden‐onset heavy vaginal bleeding that had persisted for 24 h. She had undergone a cesarean section 39 days prior due to severe preeclampsia. Her obstetric history included three chemical pregnancies and a left tubal pregnancy requiring salpingectomy. No postpartum complications or sexual activity were reported.

## 3 Diagnostic Workup

### 3.1 Physical Examination

A large cervical blood clot was noted on gynecological examination.

### 3.2 Laboratory Tests

Serum β‐hCG level was markedly elevated (7868 mIU/mL).

### 3.3 Imaging

**FIGURE 1:**
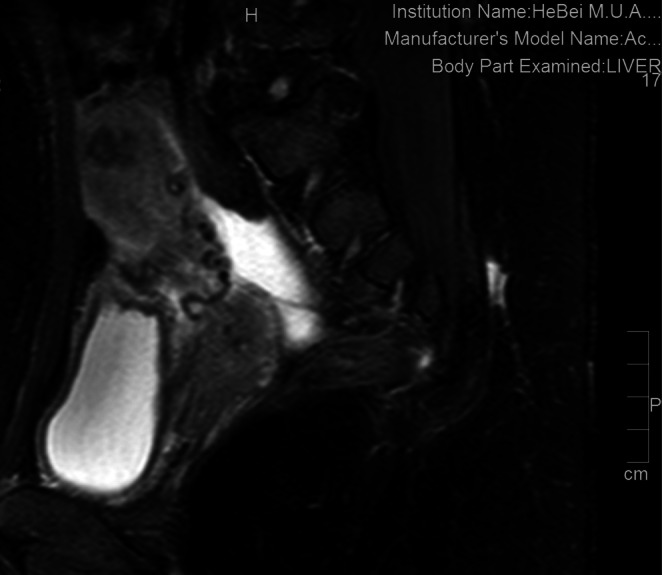
Imaging findings of the pelvis examined by MRI before treatment.

Transvaginal ultrasound revealed a vascularized uterine mass with myometrial invasion. Pelvic MRI suggested residual tissue implantation (Figure 1).

### 3.4 Procedures

Curettage removed blood clots but no pathological tissue.

## 4 Treatment and Follow‐Up

**FIGURE 2:**
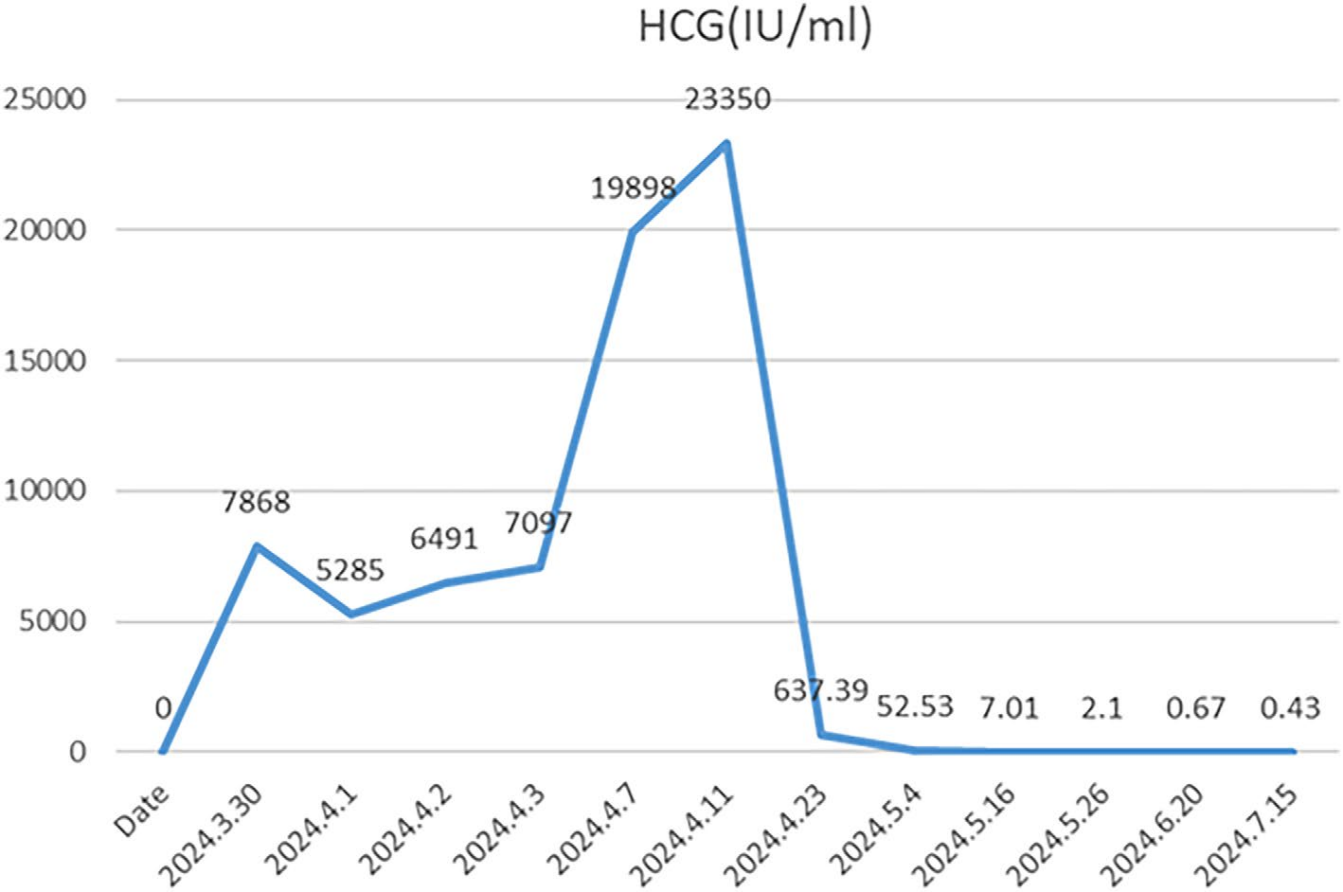
The changes in β‐human chorionic gonadotropin (β‐hCG) before diagnosis to the end of all treatments.

**FIGURE 3:**
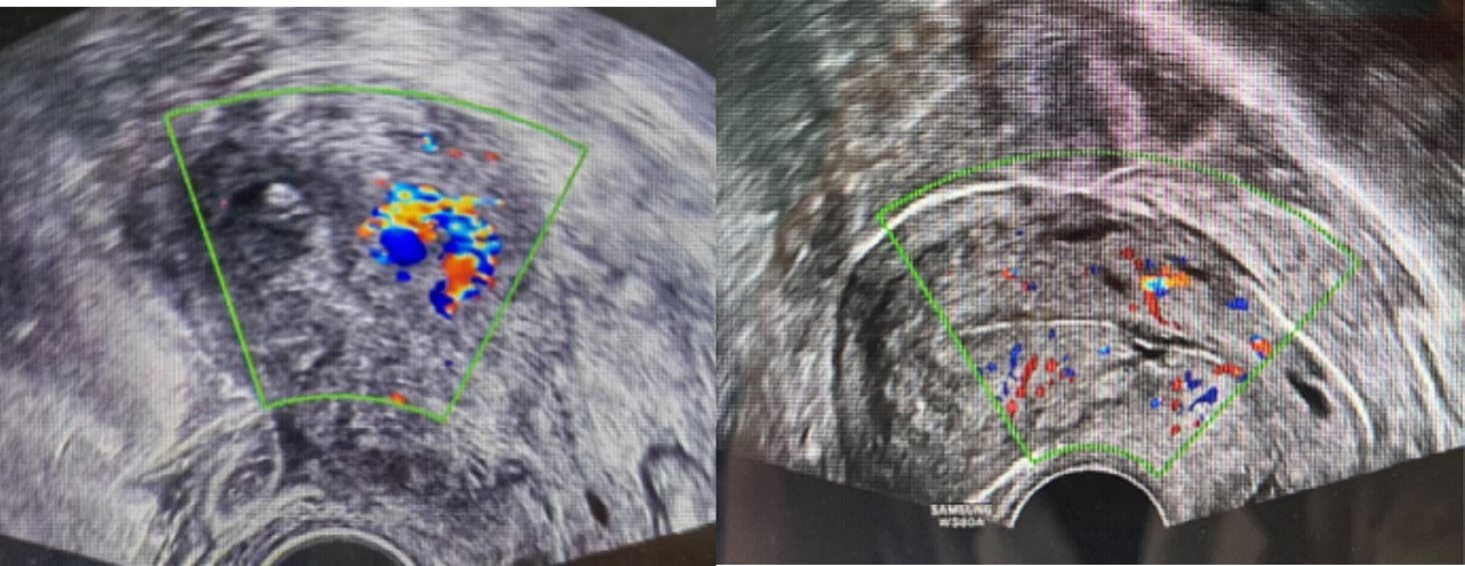
Changes in Doppler ultrasonography from pre‐diagnosis to post‐chemotherapy.

FAV chemotherapy (fluorouracil + actinomycin D + vincristine) was initiated. After two cycles, β‐hCG normalized, and imaging lesions resolved (Figures 2 and 3). Three consolidation cycles were completed by July 20, 2024. The patient remains asymptomatic at the last follow‐up, with transient chemotherapy‐related diarrhea resolving spontaneously.

## 5 Differential Diagnoses

Persistent postpartum hemorrhage necessitates the exclusion of common etiologies. In this case, retained placental fragments were initially suspected due to elevated β‐hCG and imaging findings. However, curettage yielded no placental tissue, and β‐hCG trends (initial decline followed by a rebound) contradicted retained products. Uterine arteriovenous malformation was also considered but ruled out by the absence of characteristic Doppler findings (e.g., turbulent flow) [10]. Additionally, hormonal causes (e.g., pituitary dysfunction) were excluded via endocrine profiling. The combination of β‐hCG dynamics, imaging features, and exclusion of alternative diagnoses supported the diagnosis of choriocarcinoma.

## 6 Discussion

Choriocarcinoma, a malignancy that can arise secondarily from various pregnancy outcomes, including miscarriage, ectopic pregnancy, and intrauterine gestation, represents an exceedingly uncommon sequela of childbirth, with an incidence rate approximating one in 50 000 deliveries [4]. In its early stages, postpartum choriocarcinoma lacks distinctive clinical presentations, often manifesting solely as irregular vaginal bleeding, which can lead to misdiagnosis or delayed recognition. Many instances of postpartum irregular vaginal bleeding are erroneously attributed to factors such as suboptimal uterine involution or retained placental fragments, thereby obscuring the possibility of choriocarcinoma and delaying timely diagnosis and intervention. Consequently, the prognosis for postpartum choriocarcinoma tends to be more dire compared to its counterparts arising after other pregnancy‐related events, emphasizing the critical need for heightened vigilance and a comprehensive diagnostic approach.

The latency period of postpartum choriocarcinoma varies significantly. A retrospective study by Nugent et al. (2006) involving 35 patients found that the average time from the onset of symptoms to diagnosis ranged between 7 and 19 weeks postpartum [2]. Similarly, a retrospective cohort study conducted at Peking Union Medical College Hospital, which analyzed the clinical characteristics and prognosis of 272 patients with postpartum placental choriocarcinoma, reported the longest interval between the previous pregnancy and diagnosis to be 29 years. Nearly half of the patients in this study had an interval of more than 12 months from their first pregnancy to diagnosis [11]. These findings indicate that choriocarcinoma after full‐term delivery is clinically rare, with a variable latency period, resulting in a relatively high misdiagnosis rate.

This case exemplifies the diagnostic challenges of postpartum choriocarcinoma. Despite inconclusive placental pathology—a common pitfall in routine practice—rapid diagnosis was achieved through vigilant β‐hCG trend analysis (Figure 2) and interdisciplinary consensus between obstetrics, radiology, and oncology teams. Notably, the absence of placental abnormalities contrasts with previous reports where histopathology was definitive, highlighting the need for clinical suspicion even when pathological evidence is lacking [6]. Emerging techniques such as circulating tumor DNA analysis or PET‐MRI fusion imaging may further enhance diagnostic precision in equivocal cases [12, 13, 14].

In the early stages, postpartum choriocarcinoma often lacks overt symptoms but exhibits a marked propensity for disseminating to vital organs such as the liver and brain [15, 16, 17, 18, 19]. Clinical recognition of this malignancy typically occurs when patients exhibit indicators of metastatic disease, including abnormal vaginal bleeding, respiratory distress manifested by cough and hemoptysis stemming from lung metastases, and neurological disturbances like headaches and hemiplegia suggestive of brain involvement. Research consistently highlights the lungs as the primary site of metastatic spread in choriocarcinoma, accounting for approximately 89% of cases [20], with notable proportions also affecting the liver and brain [5]. This emphasizes the imperative for heightened vigilance and expedited diagnostic evaluations.

It is crucial to emphasize that bleeding from metastatic sites, particularly when intracranial hemorrhages occur, can pose a grave, life‐threatening risk. Given that metastatic symptoms frequently constitute the primary reason for postpartum women to seek medical attention, they often initially consult non‐gynecological specialists. This contributes to a high incidence of misdiagnosis due to the rarity of choriocarcinoma. In this specific case study, the patient presented with irregular vaginal bleeding but lacked overt metastatic lesions at the time of diagnosis, owing to timely detection and referral. This posed a unique diagnostic challenge, underscoring the importance of maintaining a broad differential diagnosis in such scenarios and highlighting the value of interdisciplinary collaboration in achieving accurate and timely diagnoses.

The cornerstone of diagnosing postpartum choriocarcinoma, especially in cases of postpartum hemorrhage, is the assessment of serum β‐hCG concentrations. Typically, these levels regress to normal ranges within 1–3 weeks post‐delivery in uncomplicated scenarios. Conversely, when gestational tissues are retained, normalization of serum β‐hCG may be protracted, potentially extending up to 21–35 days following delivery. As such, once the possibility of retained products of conception or a subsequent gestation has been diligently excluded through comprehensive investigations, a definitive diagnosis of choriocarcinoma can be confidently made, based on the sustained presence of abnormally elevated serum β‐hCG levels [21].

While the diagnosis of GTN primarily relies on the patient's clinical history and β‐hCG levels, histopathological analysis and imaging studies play a crucial role in facilitating an accurate early diagnosis [1, 5, 22]. The hallmark pathological feature of choriocarcinoma is the infiltration of syncytiotrophoblasts and cytotrophoblasts into the myometrium [5]. Mere visual inspection of the postpartum placenta often falls short in detecting abnormalities, necessitating histopathological evaluation to confirm the diagnosis. Unfortunately, routine histopathological evaluation of the placenta during normal deliveries is uncommon, leading to a low detection rate of postpartum choriocarcinoma. Therefore, it is imperative to prioritize thorough visual examination of the placenta, particularly in high‐risk cases, and to proceed with histopathological analysis whenever abnormalities are detected. In this specific case, although placental examination was conducted post‐cesarean delivery, postnatal placental pathology surprisingly revealed no abnormalities. Curettage or hysteroscopy can be invaluable in controlling bleeding and obtaining biopsy specimens for histopathological evaluation. However, acquiring pathological results can prove challenging, as exemplified in this case report, where the hemorrhagic nature of the lesion hindered the retrieval of pathological tissue. Despite this, vigilant monitoring of serum β‐hCG levels remains indispensable, even in instances where pathological findings are inconclusive.

Furthermore, transvaginal ultrasonography serves as a crucial diagnostic tool for choriocarcinoma. The typical presentation of this malignancy includes a highly vascularized mass characterized by myometrial invasion and poorly defined margins. Remarkably, choriocarcinoma is a disease that can often be fully cured through chemotherapy, with a remarkable cure rate of up to 90% [5]. Currently, it is widely believed that carrying a pregnancy to full term may significantly increase the risk of a poor prognosis, except in certain circumstances, such as when the initial hCG level surpasses 100 000 IU/24 h before treatment, the time between the end of pregnancy and the commencement of treatment exceeds 4 months, or there is a history of unsuccessful chemotherapy, brain metastases, or liver metastases [23, 24]. Notably, postpartum choriocarcinoma often exhibits poor responsiveness to single‐drug chemotherapy. Consequently, the recommended treatment approach for postpartum choriocarcinoma, regardless of the World Health Organization prognostic score, involves the use of multi‐drug chemotherapy [5, 23, 25]. Presently, commonly utilized chemotherapy regimens include EMA‐CO, EMA/EP, BEP, among others. It is essential to monitor hCG variations and tumor shrinkage during chemotherapy to assess treatment efficacy and adjust the chemotherapy regimen if needed, as inadequate regimens may lead to drug resistance and compromise the prognosis.

## 7 Conclusion

In conclusion, this study underscores the importance of heightened vigilance and a comprehensive diagnostic approach in identifying postpartum choriocarcinoma, given its rare occurrence, variable latency period, and high misdiagnosis rate. The findings emphasize the significance of serum β‐hCG monitoring, histopathological analysis, and interdisciplinary collaboration in achieving accurate and timely diagnoses. Clinically, these insights have substantial value in guiding prompt and effective treatment strategies, particularly the use of multi‐drug chemotherapy, to improve patient outcomes and prognosis.

## Author Contributions

Z.D., S.W., L.S. and S.Z. contributed to patient care, the conception of the case report, the acquisition and interpretation of data, and the drafting and critical revision of the article for important intellectual content. All authors approved the final submitted manuscript.

## Consent

The patient provided written, informed consent for the publication of this report and the accompanying images.

## Conflicts of Interest

The authors declare no conflicts of interest.

## Data Availability

The data that support the findings of this study are available from the corresponding author upon reasonable request.

## References

[1] G. L. Dai , F. R. Tang , Y. Ma , and D. Q. Wang , “Postpartum Choriocarcinoma–A Rare Cause of Delayed Postpartum Hemorrhage: Four Case Reports and Literature Review,” Medicine (Baltimore) 103, no. 11 (2024): e37510. 38489712 10.1097/MD.0000000000037510 PMC10939666

[2] D. Nugent , A. Hassadia , J. Everard , B. W. Hancock , and J. A. Tidy , “Postpartum Choriocarcinoma Presentation, Management and Survival,” Journal of Reproductive Medicine 51, no. 10 (2006): 819–824. 17086810

[3] J. Li , J. Yang , P. Liu , et al., “Clinical Characteristics and Prognosis of 272 Postterm Choriocarcinoma Patients at Peking Union Medical College Hospital: A Retrospective Cohort Study,” BMC Cancer 16 (2016): 347. 27251425 10.1186/s12885-016-2383-1 PMC4890243

[4] J. T. Soper , “Gestational Trophoblastic Disease: Current Evaluation and Management,” Obstetrics and Gynecology 137, no. 2 (2021): 355–370. 33416290 10.1097/AOG.0000000000004240 PMC7813445

[5] T. Chawla , G. Bouchard‐Fortier , G. Turashvili , R. Osborne , K. Hack , and P. Glanc , “Gestational Trophoblastic Disease: An Update,” Abdominal Radiology (NY) 48, no. 5 (2023): 1793–1815. 10.1007/s00261-023-03820-5 36763119

[6] T. L. Mao and I. Shih , “Advances in the Diagnosis of Gestational Trophoblastic Tumors and Tumor‐Like Lesions,” Expert Opinion on Medical Diagnostics 3, no. 4 (2009): 371–380. 23485206 10.1517/17530050903032646

[7] J. A. Tidy , G. J. S. Rustin , E. S. Newlands , et al., “Presentation and Management of Choriocarcinoma After Nonmolar Pregnancy,” British Journal of Obstetrics and Gynaecology 102, no. 9 (1995): 715–719. 7547762 10.1111/j.1471-0528.1995.tb11429.x

[8] M. C. Macdonald , R. Ram , J. A. Tidy , and B. W. Hancock , “Choriocarcinoma After a Nonterm Pregnancy,” Journal of Reproductive Medicine 55, no. 5–6 (2010): 213–218. 20626177

[9] W. Ding , N. Zhang , Y. Rao , X. Xu , T. Nie , and P. Qu , “A Successfully Treated Multiple Metastatic Choriocarcinoma Coexistent With Live Fetus: A Case Report and Literature Review,” Frontiers in Oncology 11 (2021): 777707. 35174067 10.3389/fonc.2021.777707 PMC8841587

[10] C. A. Keller , N. Antil , R. B. Jeffrey , and A. Kamaya , “Color Doppler Imaging of Vascular Abnormalities of the Uterus,” Ultrasound Quarterly 38, no. 1 (2022): 72–82. 35239631 10.1097/RUQ.0000000000000578

[11] M. I. Bianconi , S. Otero , O. Moscheni , L. Alvarez , C. Storino , and G. Jankilevich , “Gestational Trophoblastic Disease: A 21‐Year Review of the Clinical Experience at an Argentinean Public Hospital,” Journal of Reproductive Medicine 57, no. 7–8 (2012): 341–349. 22838252

[12] K. Ravn , L. Hatt , R. Singh , et al., “Diagnosis of Hydatidiform Moles Using Circulating Gestational Trophoblasts Isolated From Maternal Blood,” Placenta 135 (2023): 7–15. 36889013 10.1016/j.placenta.2023.02.012

[13] L. Sunde , R. Singh , K. Ravn , et al., “Hydatidiform Mole Diagnostics Using Circulating Gestational Trophoblasts Isolated From Maternal Blood,” Molecular Genetics & Genomic Medicine 9, no. 1 (2021): e1565. 33306861 10.1002/mgg3.1565 PMC7963416

[14] M. R. Openshaw , R. A. Harvey , N. J. Sebire , et al., “Circulating Cell Free DNA in the Diagnosis of Trophoblastic Tumors,” eBioMedicine 4 (2016): 146–152. 26981554 10.1016/j.ebiom.2015.12.022 PMC4776063

[15] M. Tripathi , M. M. D'Souza , J. Jain , et al., “Metastatic Choriocarcinoma With Tumor Thrombus in the Right Atrium and Pulmonary Vessels: Diagnosis and Therapy Monitoring With F‐18 Flurodeoxyglucose PET/CT,” Clinical Nuclear Medicine 34, no. 6 (2009): 381–385. 19487853 10.1097/RLU.0b013e3181a3461f

[16] A. Lemanska , P. Banach , K. Stanisławska , R. Juszkat , M. Spaczyński , and E. Nowak‐Markwitz , “Urgent Embolization of Hemorrhagic Choriocarcinoma Liver Metastases–Case Report and Review of the Literature,” Ginekologia Polska 86, no. 12 (2015): 957–961. 26995948 10.17772/gp/57871

[17] K. Fetcko and M. Dey , “Primary Central Nervous System Germ Cell Tumors: A Review and Update,” Medical Research Archives 6, no. 3 (2018): 1719. 30271875 10.18103/mra.v6i3.1719 PMC6157918

[18] H. Ngan , M. J. Seckl , R. S. Berkowitz , et al., “Diagnosis and Management of Gestational Trophoblastic Disease: 2021 Update,” International Journal of Gynecology & Obstetrics 155, no. Suppl 1 (2021): 86–93. 10.1002/ijgo.13877 PMC9298230 34669197

[19] A. Akhavan‐Sigari , Y. S. Hori , P. M. Harary , et al., “Stereotactic Radiosurgery for Choriocarcinoma Brain Metastases: Illustrative Case Presentation and Systematic Review,” World Neurosurgery 194 (2025): 123387. 39491621 10.1016/j.wneu.2024.10.116

[20] L. S. Dobson , A. M. Gillespie , R. E. Coleman , and B. W. Hancock , “The Presentation and Management of Post‐Partum Choriocarcinoma,” British Journal of Cancer 79, no. 9–10 (1999): 1531–1533. 10188902 10.1038/sj.bjc.6690244 PMC2362714

[21] K. J. Rodabaugh , M. R. Bernstein , D. P. Goldstein , and R. S. Berkowitz , “Natural History of Postterm Choriocarcinoma,” Journal of Reproductive Medicine 43, no. 1 (1998): 75–80. 9475153

[22] M. Mangla , E. A. Rahiman , H. Kaur , and P. Kanikaram , “Gestational Trophoblastic Neoplasia With Concurrent Metastasis to the Mother and Child: A Systematic Literature Review,” Journal of the Turkish‐German Gynecological Association 24, no. 3 (2023): 206–219. 37675557 10.4274/jtgga.galenos.2023.2023-5-2 PMC10493811

[23] G. Bogani , I. Ray‐Coquard , D. Mutch , et al., “Gestational Choriocarcinoma,” International Journal of Gynecological Cancer 33, no. 10 (2023): 1504–1514. 37758451 10.1136/ijgc-2023-004704

[24] E. Katsanevakis , A. Oatham , and D. Mathew , “Choriocarcinoma After Full‐Term Pregnancy: A Case Report and Review of the Literature,” Cureus 14, no. 2 (2022): e22200. 35308750 10.7759/cureus.22200 PMC8925935

[25] H. O. Smith , E. Kohorn , and L. A. Cole , “Choriocarcinoma and Gestational Trophoblastic Disease,” Obstetrics and Gynecology Clinics of North America 32, no. 4 (2005): 661–684. 16310678 10.1016/j.ogc.2005.08.001

